# Clinically-Inspired Multi-Agent Transformers for Disease Trajectory Forecasting From Multimodal Data

**DOI:** 10.1109/TMI.2023.3312524

**Published:** 2024-01-02

**Authors:** Huy Hoang Nguyen, Matthew B. Blaschko, Simo Saarakkala, Aleksei Tiulpin

**Affiliations:** Research Unit of Health Sciences and Technology, University of Oulu, 90220 Oulu, Finland; Center for Processing Speech and Images, KU Leuven, 3000 Leuven, Belgium; Research Unit of Health Sciences and Technology, University of Oulu, 90220 Oulu, Finland; Department of Diagnostic Radiology, Oulu University Hospital, 90220 Oulu, Finland; Research Unit of Health Sciences and Technology, University of Oulu, 90220 Oulu, Finland; Neurocenter Oulu, Oulu University Hospital, 90220 Oulu, Finland

**Keywords:** Deep Learning, knee, osteoarthritis, prognosis prediction

## Abstract

Deep neural networks are often applied to medical images to automate the problem of medical diagnosis. However, a more clinically relevant question that practitioners usually face is how to predict the future trajectory of a disease. Current methods for prognosis or disease trajectory forecasting often require domain knowledge and are complicated to apply. In this paper, we formulate the prognosis prediction problem as a one-to-many prediction problem. Inspired by a clinical decision-making process with two agents–a radiologist and a general practitioner – we predict prognosis with two transformer-based components that share information with each other. The first transformer in this framework aims to analyze the imaging data, and the second one leverages its internal states as inputs, also fusing them with auxiliary clinical data. The temporal nature of the problem is modeled within the transformer states, allowing us to treat the forecasting problem as a multi-task classification, for which we propose a novel loss. We show the effectiveness of our approach in predicting the development of structural knee osteoarthritis changes and forecasting Alzheimer’s disease clinical status directly from raw multi-modal data. The proposed method outperforms multiple state-of-the-art baselines with respect to performance and calibration, both of which are needed for real-world applications. An open-source implementation of our method is made publicly available at https://github.com/Oulu-IMEDS/CLIMATv2.

## I. Introduction

RECENT developments in Machine Learning (ML) suggest that it is soon to be tightly integrated into many fields, including healthcare [1], [2]. One particular subfield of ML – Deep Learning (DL) has advanced the most, as it opened the possibility to make predictions from high-dimensional data. In medicine, this impacted the field of radiology, in which highly trained human readers identify pathologies in medical images. The full clinical pipeline, however, aims to assess the condition of a patient as a whole, and eventually prescribe the most relevant treatment for a disease [3], [4]. Using DL in this broad scope by integrating multimodal data has the potential to provide even further advances in medical applications.

Clinical diagnosis is made by specialized treating physicians or general practitioners. These doctors are not radiologists and rather use the services of the latter in decision-making. One of the typical problems that such doctors face is to make a prognosis [5], [6], which can be formalized as disease trajectory forecasting (DTF). This is an especially relevant task in degenerative disorders, often seen in musculoskeletal and nervous systems. This work studies DTF for knee osteoarthritis (OA) – the most common musculoskeletal disorder [7], and Alzheimer’s disease (AD) – the leading cause of dementia [8].

Among all the joints in the body, OA is mostly prevalent in the knee. Knee OA is characterized by the appearance of osteophytes, and the narrowing of joint space [9], which in the clinical setting are usually imaged using X-ray (radiography) [10]. The disease severity is graded according to the Kellgren-Lawrence system [11] from 0 (no OA) to 4 (end-stage OA), or Osteoarthritis Research Society International (OARSI) atlas criteria [12]. Unfortunately, OA is progressive over time (see Figure 2) and no cure has yet been developed for OA. However, diagnosing OA at an early stage may allow the slowing down of the disease, for example using behavioral interventions [13].

Individuals with AD have difficulties with reading, learning, and even performing daily activities. AD is fatally progressive and caused more than 120, 000 deaths in the United States in 2019; however, no effective cure for it has been made available [8]. The benefits of early AD diagnosis are similar to OA – the progression of the disease can be delayed, and patients may be assigned relevant care in a timely manner [14].

In both of the aforementioned fields – OA and AD, there is a lack of studies on prognosis prediction. From an ML perspective, a more conventional setup is to predict *whether* the patient has the disease at present or a specific point of time in the future [15], [16], [17], [18], [19], [20], [21]. However, prognosis prediction aims to answer *whether* and *how* the disease would evolve over time. Furthermore, in a real-life situation, the treating physician makes the prognosis while interacting with a radiologist or other stakeholders who can provide information (e.g. blood tests or radiology reports) about the patient’s condition. This also largely differentiates the diagnostic task from predicting a prognosis.

**Fig. 1.**
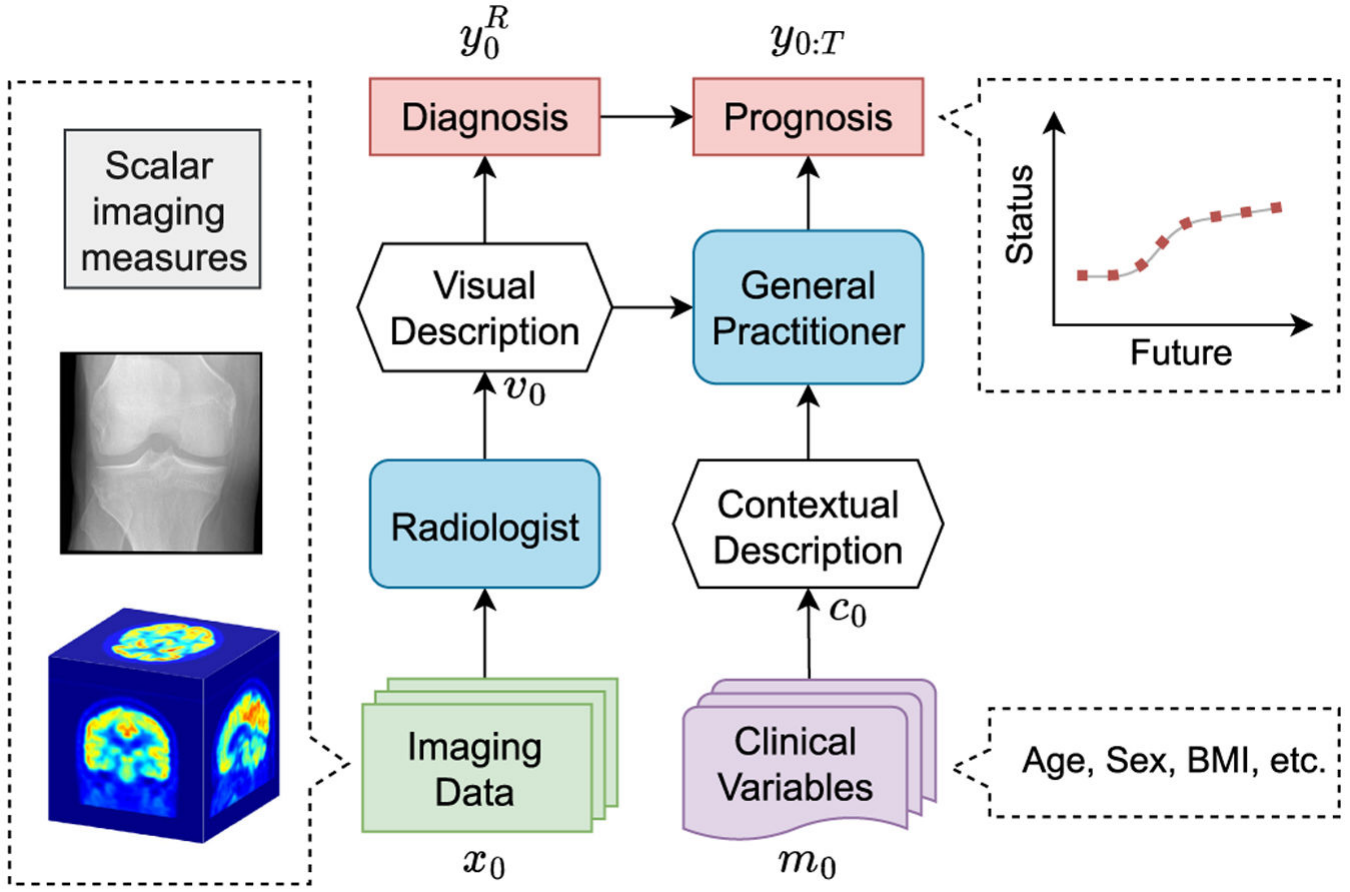
The concept of CLIMATv2 was inspired by a multi-agent decision-making system with a radiologist and a general practitioner. All types of imaging data of disease are handled by the radiologist. The general practitioner then utilizes a report description produced by the radiologist and the context of clinical variables to forecast a future trajectory of the disease.

**Fig. 2.**
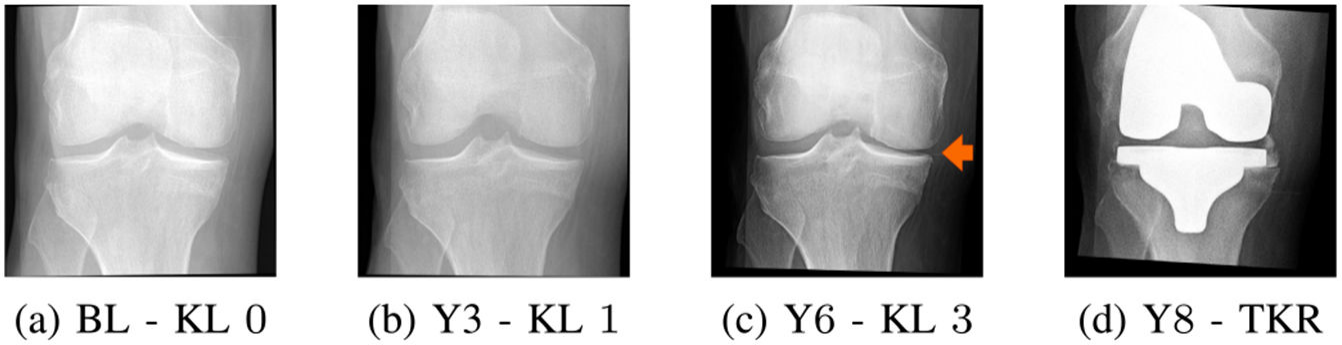
Radiographs of a patient with knee OA progressed in 8 years. The orange arrow indicates joint space narrowing. The disease progressed from Kellgren-Lawrence (KL) grade 0 at the baseline (BL) to 3 in 6 years. At the 8th year, the patient underwent a total knee replacement (TKR) surgery.

In this paper, we present an extended version of our earlier work on automatic DTF [22], where we proposed a Clinically-Inspired Multi-Agent Transformers (CLIMAT) framework, aiming to mimic the interaction process between a general practitioner / treating physician^1^ and a radiologist. In our system, a radiologist module, consisting of a feature extractor (convolutional neural network; CNN) and a transformer, analyses the input imaging data and then provides an output state of the transformer representing a radiology report to the general practitioner – corresponding module (purely transformer-based). The latter fuses this information with auxiliary patient data, and makes the prognosis prediction. We graphically illustrate the described idea in Figure 1.

Compared to the conference version [22], we have enhanced our framework, such that the module corresponding to the general practitioner does not only perform prognosis, but is also encouraged to make diagnostic predictions consistent with a radiologist module. The earlier version of CLIMAT relies on a simplifying assumption in relation to the independence between the diagnostic label task and non-imaging data. The introduced update helps the framework to expand out of the knee osteoarthritis domain, and be more realistic, thereby allowing our method to be applied in fields where diagnosis could rely on both imaging and non-imaging data. Moreover, we equip the framework with a new loss – Calibrated Loss based on Upper Bound (CLUB) – that aims to maintain the performance while improving the calibration of the framework’s predictions. Finally, we have also expanded the application of our framework to the case of AD.

To summarize, our contributions are the following:

1. We propose CLIMATv2, a clinically-inspired transformer-based framework that can learn to forecast disease severity from multi-modal data in an end-to-end manner. The main novelty of our approach is the incorporation of prior knowledge of the decision-making process into the model design.
2. We derive the CLUB loss, an upper bound on a temperature-scaled cross-entropy (TCE), and apply it to the DTF problem we have at hand. Experimentally, we show that CLUB provides better calibration and yields similar or better balanced accuracy than the competitive baselines.
3. From a clinical perspective, our results show the feasibility to perform fine-grained prognosis of knee OA and AD directly from raw multi-modal 2D and 3D data.

## II. Related Work

### A. Knee Osteoarthritis Prognosis

The attention of the literature has gradually been shifting from diagnosing the current OA severity of a knee to predicting whether degenerative changes will happen within a specified time frame. While some studies [15], [16], [17] aimed to predict whether knee OA progresses within a specified duration, others [18], [19] tried to predict if a patient will undergo a total knee replacement (TKR) surgery at some point in the future. However, the common problem of the aforementioned studies is that the scope of knee OA progression is limited to a single period of time or outcome, which substantially differentiates our work from the prior art.

### B. Alzheimer’s Disease Prognosis

Compared to the field of OA, a variety of approaches have been proposed to process longitudinal data in the AD field. Lu et al. [21] utilized a fully-connected network (FCN) to predict AD progression within a time frame of 3 years from magnetic resonance imaging (MRI) and fluorodeoxyglucose positron emission tomography (FDG-PET) scans. Ghazi et al. [23] and Jung et al. [20] used different long-short-term memory (LSTM)-based models to predict AD clinical statuses from scalar MRI biomarkers. Albright et al. [24] took into account various combinations of scalar measures and clinical variables to predict changes in AD statuses using FCNs and recurrent neural networks (RNN). In contrast to the prior art relying on either raw imaging data or scalar measures, our method enables learning from raw imaging scans, imaging-based measurements, and other scalar variables simultaneously. Additionally, whereas FCN and sequential networks were widely used in the literature, we propose to use a transformer-based framework to perform the AD clinical status prognosis task. Furthermore, we use FCN, two well-known sequential models – gated recurrent unit (GRU) and LSTM – as our reference approaches.

### C. Transformers for Vision Tasks

Although originally developed in the field of natural language processing [25], [26], transformer-based architectures have recently been applied also in vision tasks. Dosovitskiy et al. [27] pioneered the use of transformer-based architectures without a CNN for image classification problems. Girdhar et al. [28] and Arnab et al. [29] studied the same family of architectures to perform video recognition tasks. However, Hassani et al. [30] pointed out such pure transformers require a significantly large amount of imaging data to perform well. The reason is that transformers do not have well-informed inductive biases, which are strengths of CNNs. Thus, our method relies on [30] due to medium dataset sizes.

### D. Multimodal Data Processing With Transformers

Transformers have been empirically robust in learning various categories of tasks from sequential data such as text or tabular data [25], [31]. However, in medical imaging, it is common to acquire multiple modalities comprising both raw images (e.g. plain radiographs, MRI, or PET scans) and tabular data, which are challenging for a single transformer. Recent work has shown that multiple transformers are needed to for such multiple modalities [32]. Therefore, similar to our previous version [22], this study adapts the idea of using multiple transformers in our framework to perform DTF from multiple modalities.

## III. Methods

### A. The CLIMAT Framework: A Conceptual Overview

As mentioned earlier, we base our framework on multi-agent decision-making processes in a clinical setting. In many applications, this can be considered information passing between two agents – a radiologist and a general practitioner [33]. While the radiologist specializing in imaging diagnosis is in charge of producing radiology reports, the general practitioner relies on various modalities including the radiologic findings to forecast the severity of a certain disease. We model such collaboration by the concept presented in Figure 1. Specifically, the radiologist analyzes a medical image $x_{0}$ (e.g. radiograph or PET image) of a patient to provide an interpretation with rich visual description and annotations, allowing the diagnosis of the current stage $y_{0}^{R}$ of the disease. Subsequently, the general practitioner relies on (i) the clinical data $m_{0}$ (e.g. questionnaires or symptomatic assessments) with a further interpretation if needed, (ii) the provided radiology report, and (iii) the referenced diagnosis of the radiologist $y_{0}^{R}$ to predict the course of the disease $y_{0:T}$.

**Fig. 4.**
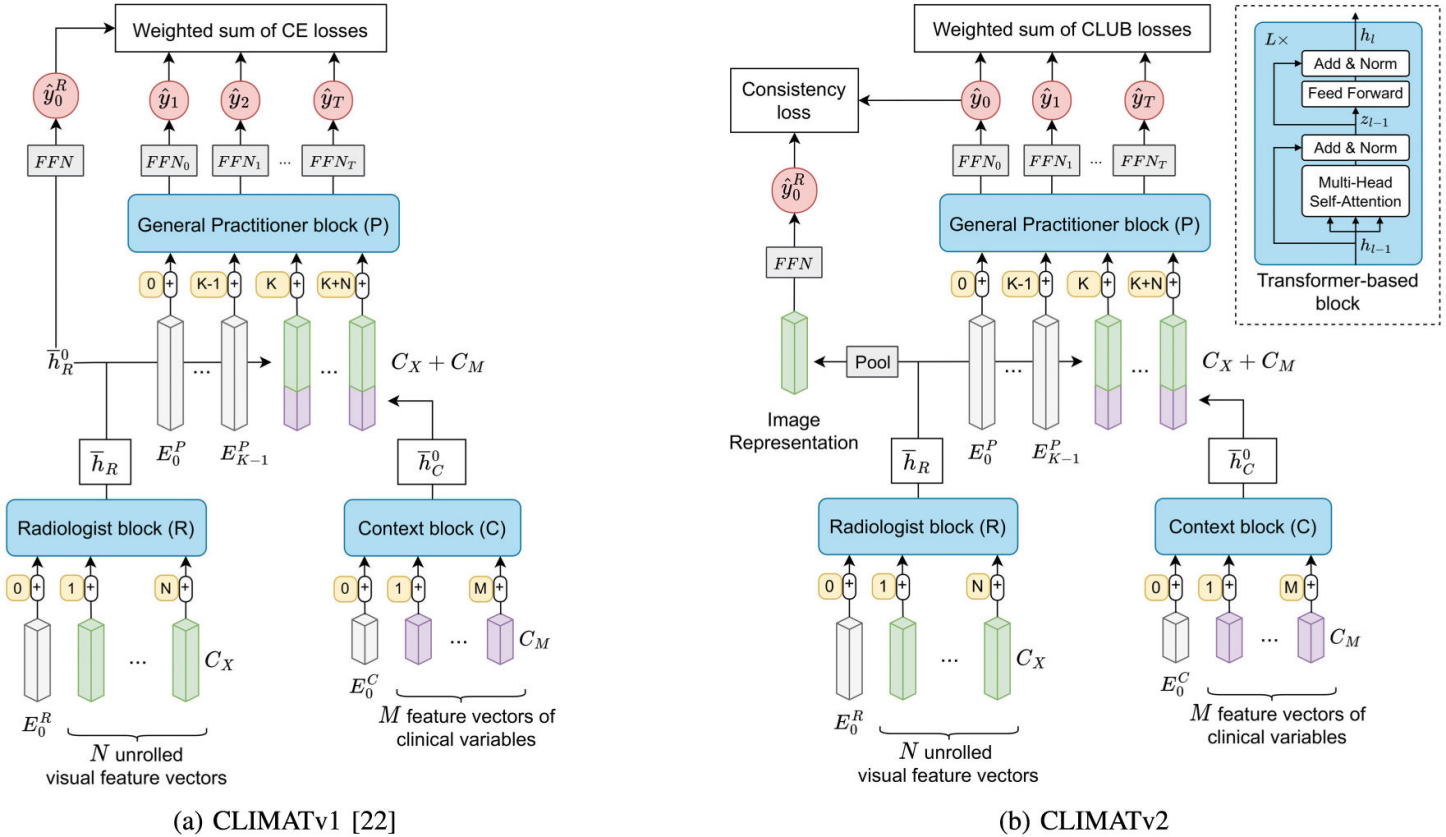
The CLIMAT framework (best viewed in color). There are N and M input imaging and non-imaging feature vectors, respectively. The first feature vector ${\bar{h}}_{C}^{0}$ of the last layer of the transformer C is appended to every output vector of ${\bar{h}}_{R}$ to form the input for the transformer P All the blue blocks are transformer-based networks. [*CLS*] and [*POS*] embeddings are in white and orange, respectively.

We implement the concept proposed above in the CLIMATv2 framework (see Figure 4 and Section III-B). CLIMATv2 comprises three primary transformer-based blocks–^2^namely Radiologist (R), Context (C), and General Practitioner (P). Firstly, assume that we obtain visual features learned from the imaging data $x_{0}$. Then, the block R acts as the radiologist to perform visual reasoning from the visual features and predict the current stage ${\hat{y}}_{0}^{R}$ of a disease. The other two blocks are responsible for context extracting and prognosis predicting. As such, the block C aims to extract a context embedding from clinical variables $m_{0}$. Subsequently, the block P utilizes the combination of the context embedding and the output states of the block R to forecast the disease trajectory ${\hat{y}}_{0:T}$.

In this work, we have two major upgrades to CLIMATv1 [22]. Firstly, we do not assume anymore that $y_{0}$ and $m_{0}$ are independent, as this does not hold in many medical imaging domains, e.g. for OA [34]. Namely, in the current version of CLIMAT, both the blocks R and P have now been allowed to make diagnosis predictions simultaneously, making sure that the learned embeddings contain information on $y_{0}$. Furthermore, we encourage their predictions to be consistent with the final module of our model. Secondly, besides performance, in this work, we take into account model calibration, which allows us to gain better insights into the reliability of models’ predictions [35]. To facilitate better calibration within our proposed framework, we propose a novel loss, called CLUB, presented in Section III-C.

### B. Technical Realization

#### 1) Transformer:

A transformer encoder comprises a stack of L multi-head self-attention layers, whose input is a sequence of vectors ${\left\{s_{i}\right\}}_{i=1}^{N}$ where $s_{i}\in R^{1\times C}$, and C is the feature size. As such, a transformer is formulated as [25]

(1) $$h_{0}=[E_{\left[CLS\right]},s_{1},\ldots ,s_{N}]+E_{\left[POS\right]},$$

(2) $$z_{l-1}=\text{MSA}(\text{LN}(h_{l-1}))+h_{l-1},$$

(3) $$h_{l}=\text{MLP}(\text{LN}(z_{l-1}))+z_{l-1},\;\;l=\{1,\ldots ,L\}$$

(4) $$\bar{h}=h_{L}$$

where $E_{\left[CLS\right]}$ is a learnable token, $E_{\left[POS\right]}$ is a learnable positional embedding, and $\bar{h}$ represents features extracted from the last layer. MLP is a multi-layer perceptron (i.e. a fully-connected network), LN is the layer normalization [36], and MSA(·) is a multi-head self-attention (MSA) layer [25]. The self-attention mechanism relies on the learning of query, key, value parameter matrices, denoted by $W_{l}^{Q}$, $W_{l}^{K}$, and $W_{l}^{V}$ with l=1,…,L, respectively. Initially, we simultaneously set $Q_{0}$, $K_{0}$, and $V_{0}$ to $h_{0}$ defined in Eq. (1). When iterating through layers l=1,…,L, we update the states as follows

(5) $$Q_{l}=Q_{l-1}W_{l}^{Q}\;K_{l}=K_{l-1}W_{l}^{K}\;V_{l}=V_{l-1}W_{l}^{V}$$

Finally, the self-attention is established thanks to the *scaled dot-product* function applied to $Q_{l}$, $K_{l}$, and $V_{l}$, and defined as

(6) $$\text{Attention}(Q_{l},K_{l},V_{l})=\text{Softmax}\left(\frac{Q_{l}K_{l}^{⊺}}{\sqrt{d_{k}}}\right)V_{l},$$

where $d_{k}$ is the feature dimension of $Q_{l}$. In essence, $Q_{l}K_{l}^{⊺}$ represents the association between all pairs of queries and keys. The normalization based on $d_{k}$ is critical to address the case where the magnitude of entries in $Q_{l}K_{l}^{⊺}$ is too large. The essential part that produces the attention is the utilization of softmax, which allows for the creation of a normalized heatmap over the association of $Q_{l}$ and $K_{l}$. Subsequently, by adding more sets of learnable weights $W_{l}^{Q}$, $W_{l}^{K}$, and $W_{l}^{V}$, we can obtain MSA by concatenating different output heads of attention. Precisely, the MSA mechanism is formulated as follows

$$\;{\text{head}}_{l}^{h}=\text{Attention}(Q_{l},K_{l},V_{l}),\;h=1,\ldots ,H\;\text{MSA}(\cdot )=\text{Concat}({\text{head}}_{l}^{1},\ldots ,{\text{head}}_{l}^{H})W_{l}^{O},$$

where H is the number of heads, and $W_{l}^{O}$ represents learning parameters associated with the H output heads.

The three main blocks in our framework are transformer-based networks (see Figure 4). While the blocks R and C have only 1 [*CLS*] token, the block P can include *K* [*CLS*] tokens to allow for multi-target predictions. The hyperparameter K is introduced in the block P to ensure that there are enough output heads for multi-task predictions. We typically set *K* to 1 or *T* + 1. In the case *K* = *T* + 1, each output head has a corresponding [*CLS*] token.

#### 2) Multimodal Feature Extraction:

Our framework is able to handle multimodal imaging and non-imaging data. As input data can be clinical variables, raw images (i.e. 2D or 3D images), and biomarkers extracted by human experts or specialized software, we have distinct feature extraction modules for different input formats. Specifically, we use the feed-forward network (FFN), 2D-CNN, and 3D-CNN-based architectures for scalar or 1D inputs, 2D, and 3D images, respectively. As such, we pre-define common feature lengths $C_{X}$ and $C_{M}$ for all imaging and non-imaging embeddings, respectively. Each FFN-based feature extractor consists of a linear layer, a GELU activation [37], layer normalization [36], and has an output shape of $1\times C_{X}$ or $1\times C_{M}$ depending on the type of input data. In the CNN-based modules, we first unroll their output feature maps into sequences of feature vectors per image super-pixel or super-voxel, then linearly project them into a $C_{X}$-dimensional space.

#### 3) Radiologist Module:

The Radiologist block is a transformer network with $L_{R}$ layers and is responsible for processing all imaging features previously extracted in Section III-B.2. For the input data preparation, we concatenate all features of different imaging modalities to form a sequence of length N that contains $C_{X}$-dimensional image representations. Subsequently, we propagate this sequence through the transformer R. To this end, the visual embedding ${\bar{h}}_{R}\in R^{(N+1)\times C_{X}}$ produced by its last layer serves two purposes: representing radiology reports and visual features for diagnosis predictions. For the former, we subsequently combine ${\bar{h}}_{R}$ with non-imaging embeddings to constitute inputs for the General Practitioner block (see Section III-B.5). For the latter, following a common practice in [38], [39], and [40], we perform an average pooling onto ${\bar{h}}_{R}$ to generate a $C_{X}$-dimensional vector. Afterward, we pass the resulting vector through an FFN comprised of a linear layer, a GELU activation [37], and a layer normalization [36] to predict the current stage $y_{0}^{R}$ of the disorder (see Figure 4).

#### 4) Clinical Context Embedding Module:

Here, we aim to mimic the comprehension of a general practitioner over different clinical modalities (e.g. questionnaires, extra tests, and risk factors). As such, we take a single [*CLS*] embedding followed by *M* clinical vector representations extracted in Section III-B.2 to form the input sequence for the Context block (see Figure 4). The underlying architecture of the block is a transformer-based network. After passing the input sequence through the transformer C with $L_{C}$ layers, we merely use the first feature vector ${\bar{h}}_{C}^{0}$ of the last feature maps $h_{L_{C}}$ as a common contextual token representing all the non-imaging modalities.

#### 5) General Practitioner Module:

As soon as the contextual token of length $C_{M}$ is acquired from the Context block, we concatenate N+1 copies of the token ${\bar{h}}_{C}^{0}$ into the last states ${\bar{h}}_{R}$ of the transformer R to generate a sequence of N+1 mixed feature vectors with a feature size of $C_{X}+C_{M}$. We then process the obtained sequence using Eq. (1) to have the sequence of (K+N+1) feature vectors. Here, we utilize the third transformer-based module to simulate the analysis of the general practitioner over all sources of data for prognosis predictions. Specifically, after passing the input sequence through the transformer P, we utilize the first T+1 vector representations of the last layer to forecast the disease severity trajectory (${\hat{y}}_{0},\ldots ,{\hat{y}}_{T}$). Predicting disease severity at each time point requires a common or distinct FFN, which comprises a layer normalization followed by two fully connected layers separated by a GELU activation [37].

### C. Calibrated Loss Based on Upper Bound for Multi-Task

#### 1) Motivations and Formulation:

Compared to CLIMATv1 [22], we aim to optimize not only the performance but also the calibration of our model’s predictions. As CLIMATv2 simultaneously predicts a sequence of T+1 targets with different difficulties, we treat it as a multi-task predictive model. The temporal information here is contained within the transformer states. Inspired by [41], to harmonize all the tasks, we propose the CLUB loss (abbreviated from Calibrated Loss Upper Bound). However, unlike [41], which relies on the *‘not always true’* assumption that $\frac{1}{\sigma }\sum_{c^{\prime }}\;\text{exp}\left(\frac{1}{\sigma^{2}}f_{c^{\prime }}(x)\right)={\left(\sum_{c^{\prime }}\text{exp}(f_{c^{\prime }}(x))\right)}^{\frac{1}{\sigma^{2}}}$, where σ is a noise factor and $f_{c^{\prime }}(.)$ is the $c^{\prime }$-th element of the logits produced by a parametric function f, we theoretically derive CLUB as an upper bound of temperature-scaled cross-entropy (CE) loss.

Consider the *t*-th task with t∈{0…T}, let $f_{t}={\left(f_{t,1},\ldots ,f_{t,N_{n}^{t}}\right)}^{T}\;\in \;R^{N_{c}^{t}}$ denote predicted logits of CLIMATv2 (i.e. an output of the transformer P) on task t, where $N_{c}^{t}$ is the number of classes of the *t*-th target. Let $g_{t}={\left(g_{t,1},\ldots ,g_{t,N_{c}^{t}}\right)}^{T}=\text{exp}(f_{t})$. Similar to [41], we model the affection of noise $\sigma_{t}$ onto the prediction of $y_{t}$ in the scaled form $\text{Softmax}\left(\frac{1}{\sigma_{t}^{2}+\varepsilon }f_{t}\right)$, where $\varepsilon \in R_{+}$ is needed to ensure the scaled softmax to be valid for all $\sigma_{t}\in R$. For convenience, we temporarily eliminate the t index from all notations. By denoting $\tau =\frac{1}{\sigma^{2}+\varepsilon }\in R_{+}$, we rewrite the scaled softmax as

(7) $$\text{Softmax}\;(\tau \;f)={\left(\frac{g_{1}^{\tau }}{\sum_{c^{\prime }}g_{c^{\prime }}^{\tau }},\ldots ,\frac{g_{N_{c}}^{\tau }}{\sum_{c^{\prime }}g_{c^{\prime }}^{\tau }}\right)}^{⊺}\in {\left[0,1\right]}^{N_{c}},$$

where $c,c^{\prime }$ are class indices, and $\tau \;\in \;R_{+}$ is a noise factor.

Without the loss of generality, c is assumed to be the ground truth class of a certain input x. τ is the inverse temperature that can smoothen (τ≤1) or sharpen (τ>1) predicted probabilities. Here, one can observe that ${\left(\sum_{c^{\prime }}g_{c^{\prime }}^{\tau }\right)}^{\frac{1}{\tau }}$ can be seen as an absolutely homogeneous function or an $\ell_{\tau }$-norm ${\left\|g\right\|}_{\tau }$ in a Lebesgue space, when τ belongs to (0, 1) or [1, ∞), respectively. Therefore, a TCE loss can be formulated as

(8) $${ℒ}_{\text{TCE}}=-\log\;\frac{g_{c}^{\tau }}{{\left\|g\right\|}_{\tau }^{\tau }},$$

where c is the true class. When τ=1, the TCE loss becomes the vanilla CE loss

(9) $${ℒ}_{\text{CE}}=-\log\;\frac{g_{c}}{{\left\|g\right\|}_{1}}.$$

For the purpose of improving calibration, we are interested in the case of τ∈(0,1] [35], allowing us to apply the reverse Hölder’s inequality to have ${\left\|g\right\|}_{\tau }\;\leq \;N_{c}^{(1-\tau )∕\tau }{\left\|g\right\|}_{1}$. Then, we can derive an upper bound of ${ℒ}_{\text{TCE}}$, called the CLUB loss, as

$${ℒ}_{\text{CLUB}}≜-\tau \;\log\;\frac{g_{c}}{{\left\|g\right\|}_{1}}+(1-\tau )\;\log\;N_{c}\;\;=\tau {ℒ}_{\text{CE}}+(1-\tau )\;\log\;N_{c},\;\;\tau \in [0,1],$$

where the equality holds if and only if τ=1. Unlike ${ℒ}_{\text{TCE}}$, our CLUB loss directly depends on ${\left\|g\right\|}_{1}$ rather than ${\left\|g\right\|}_{\tau }$. Eq. (10) indicates that ${ℒ}_{\text{CLUB}}$ is a convex combination between the CE loss (9) and log $\log\;N_{c}$, which takes into account the task complexity in terms of the number of classes.

#### 2) Performance and Calibration Optimization:

In our setting, we consider each $\tau_{t}$ associated with task t as a learnable parameter. As the model’s parameters θ and $\tau_{t}’s$ are independent, we can respectively derive the gradients of ${ℒ}_{\text{CLUB}}(t)$ w.r.t. θ and $\tau_{t}’s$ as follows

(10) $$\frac{\partial {ℒ}_{\text{CLUB}}(t)}{\partial \theta }=\tau_{t}\frac{\partial {ℒ}_{\text{CE}}(t)}{\partial \theta },\;\;t=0\ldots T,$$

(11) $$\frac{\partial {ℒ}_{\text{CLUB}}(t)}{\partial \tau_{t}}={ℒ}_{\text{CE}}(t)-\log\;N_{c},\;\;t=0\ldots T,$$

where ${ℒ}_{\text{CE}}(t)$ and ${ℒ}_{\text{CLUB}}(t)$ are the CE and CLUB losses on the *t*-th task, respectively. Whereas the optimization w.r.t. θ essentially aims to improve the performance of our model, learning $\tau_{t}’s$ directly impacts its calibration quality. Eqs. (10) and (11) indicate that $\tau_{t}’s$ can be seen as learnable coefficients of different tasks.

**Table:** 

Algorithm 1 Constraint of *T*_*t*_ *<* 1, *t —* 0... T
$$\begin{array}{cc} \; & \;\;\;\mathrm{Input}:T:\text{the number of future time points}\\ \; & \;\;\;\mathrm{Input}:{\left\{\sigma_{t}\right\}}_{t=0}^{T}:\text{noise parameters}\\ \; & \;\;\;\mathrm{Input}:\varepsilon \in R_{+}:\text{hyperparameter}\\ \; & \;1\;\rho_{t}\leftarrow \frac{1}{\sigma_{t}^{2}+\varepsilon }\;\;t=0\ldots T\\ \; & \;2\;\mathrm{for}\;t=0,\ldots ,T\;\mathrm{do}\\ \; & \;3\;\;\;\;{\tilde{\rho }}_{t}=\frac{\text{exp}(\rho_{t})}{\sum_{t^{\prime }=0}^{T}\text{exp}(\rho_{t^{\prime }})}\\ \; & \;4\;\mathrm{end}\\ \; & \;5\;\rho_{\max}\leftarrow \text{Max}\left({\left\{{\tilde{\rho }}_{t}\right\}}_{t=0}^{T}\right)\\ \; & \;6\;\tau_{t}\leftarrow \frac{{\tilde{\rho }}_{t}}{\rho_{\max}}\;\;\;t=0\ldots T \end{array}$$

To effectively constrain $\tau_{t}\leq 1$ and avoid a trivial solution where ∀t∈{0,…,T}, $\tau_{t}=1$, we constrain the learnable parameters ${\left\{\tau_{t}\right\}}_{t=0}^{T}$ using Algorithm 1. Specifically, Line 1 guarantees that $\rho_{t}’s$ are valid for any $\sigma_{t}’s$. Lines 2 to 4 prevent all the $\tau_{t}’s$ from converging to the obvious value 1. Lines 5 and 6 re-scales $\tau_{t}’s$ such that merely ones with the maximum values become 1. This last step is necessary to avoid $\tau_{t}’s$ values being small inversely proportionally to the number of tasks.

### D. Multi-Task Learning for Disease Trajectory Forecasting

In practice, it is highly common to have data *not* fully annotated. Thus, our framework should allow for handling missing targets by design. As such, our multi-task loss can tackle such an impaired condition with ease by using an indicator function to mask out targets without annotation. Formally, we minimize the following prognosis forecasting loss

(12) $${ℒ}_{\text{prog}}=\frac{1}{\sum_{t=0}^{T}I_{t}}\sum_{t=0}^{T}I_{t}{ℒ}_{\text{CLUB}}(t),$$

where $I_{t}$ is an indicator function for task t.

While the radiologist has strong expertise in imaging diagnosis, in relation to prognosis, the general practitioner has more advantages due to the access to multimodal data, such as the patient’s background. On the other hand, general practitioners are also able to assess images to some extent. We incorporate the corresponding prior into our learning framework by enforcing consistency in predictions between the two agents:

(13) $${ℒ}_{\text{cons}}={\left\|f_{0}^{R}-f_{0}\right\|}_{1},$$

where $f_{0}^{R}$ and $f_{0}$ indicate logits of the blocks R and P for diagnosis predictions, respectively. It is worth noting that while ${ℒ}_{\text{prog}}$ operates solely on annotated targets, ${ℒ}_{\text{cons}}$ optimizes all targets.

To optimize the whole framework, we minimize the final loss ℒ as follows

(16) $$ℒ={ℒ}_{\text{prog}}+\lambda {ℒ}_{\text{cons}},$$

where $\lambda \in R_{+}$ is a consistency regularization coefficient.

## IV. Experiments

### A. Data

**Fig. 5.**
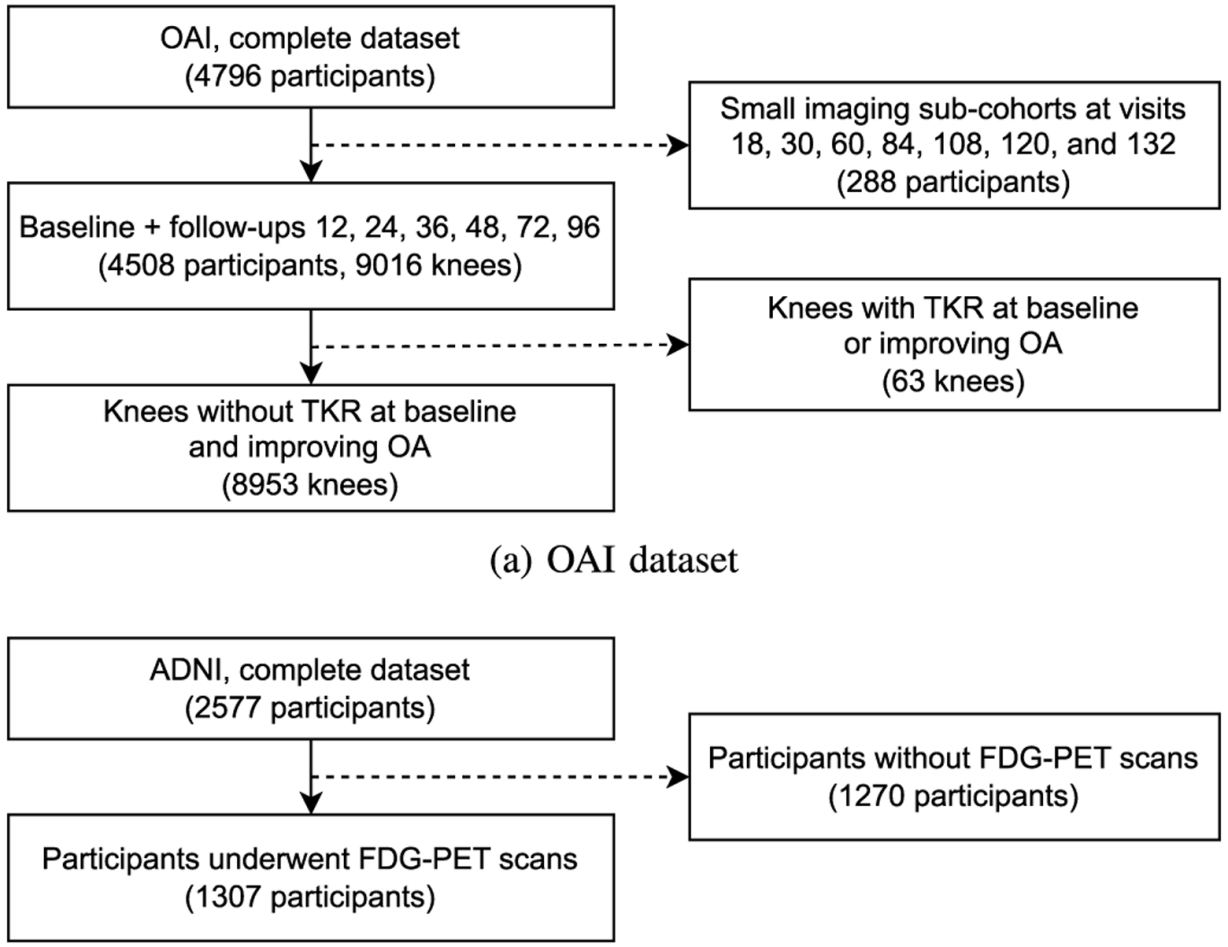
Subject selection in our study.

In this study, we conducted experiments on two public datasets for knee OA and AD. The overall description and subject selection of the two datasets and corresponding tasks can be seen in Figure 5 and Table I The details of data pre-processing and prognosis prediction tasks are presented as follows.

#### 1) Knee OA Structural Prognosis Prediction:

We conducted experiments on the Osteoarthritis Initiative (OAI) cohort, pub-icly available at https://nda.nih.gov/oai/. 4, 796 participants from 45 to 79 years old participated in the OAI cohort, which consisted of a baseline, and follow-up visits after 12, 18, 24, 30, 36, 48, 60, 72, 84, 96, 108, 120, and 132 months. In the present study, we used all knee images that were acquired with large imaging cohorts: the baseline, and the 12, 24, 36, 48, 72, and 96-month follow-ups.

As the OAI dataset includes data from five acquisition centers, we used data from 4 centers for training and validation, and considered data from the left-out one as an independent test set. On the former set, we performed a 5-fold cross-validation strategy.

Following [15] and [42], we utilized the BoneFinder tool [43] to extract a pair of knees regions from each bilateral radiograph, and pre-process each of them. Subsequently, we resized each pre-processed image to 256 × 256 pixels (pixel spacing of 0.5*mm*), and horizontally flipped it if that image corresponds to a right knee.

We utilized the Kellgren-Lawrence (KL) as well as OARSI grading systems to assess knee OA severity. The KL system classifies knee OA into 5 levels from 0 to 4, proportional to the OA severity increase. The OARSI system consists of 6 sub-systems – namely lateral/medial joint space (JSL/JSM), osteophytes in the lateral/medial side of the femur (OSFL/OSFM), and osteophytes in the lateral/medial side of the tibia (OSTL/OSTM). And according to that the furthest targets in KL, JSL, and JSM were 8 years from the baseline while it was 4 years for the other grading aspects.

Regarding the KL grading system, we grouped KL-0 and KL-1 into the same class as they are clinically similar, and added TKR knees as the fifth class. As a result, there were 5 classes in KL, and there were 4 severity levels in each of the OARSI sub-systems. Following [15] and [22], we utilized age, sex, body mass index (BMI), history of injury, history of surgery, and total Western Ontario and McMaster Universities Arthritis Index (WOMAC) as clinical variables. We quantized the continuous variables, and presented each of them by a 4-element one-hot vector depending on the relative position of its value in the interval created by the minimum and the maximum.

For clinical relevance, we did not perform knee OA prognosis predictions on knees that underwent TKR or were diagnosed with the highest grade in any OARSI sub-system. In addition, we ignored one single entry whose pair of knees were improperly localized from its lateral radiograph by the BoneFinder tool. To have more *training* samples, we generated multiple entries from the longitudinal record of each participant by considering imaging and non-imaging data at different follow-up visits (except for the last one) as additional inputs.

#### 2) AD Clinical Status Prognosis Prediction:

We applied our framework to forecast the Alzheimer’s disease (AD) clinical status from multi-modal data on the Alzheimer’s Disease Neuroimaging Initiative (ADNI) cohort, which is available at https://ida.loni.usc.edu. The recruitment was done at 57 sites around America and Canada, and there were 2, 577 male and female participants from 55 to 90 enrolled in the cohort. The participants underwent a series of tests such as clinical evaluation, neuropsychological tests, genetic testing, lumbar puncture, MRI, and PET imaging at a baseline and follow-up visits at 1, 2, and 4-year periods.

In this study, we used raw FDG-PET scans, MRI measures, cognitive tests, clinical history, and risk factors as predictor variables. The raw FDG-PET scans were pre-processed by the dataset owner, and were then standardized to voxel dimensions of 160 × 160 × 160 (1.5 × 1.5 × 1.5*mm*^3^ voxel spacing) using the NiBabel library [44]. To be in line with the OAI dataset, we applied the same technique to convert scalar inputs to one-hot encoding vectors with a length of 4. In querying subjects, while we only selected entries whose raw FDG-PET scans were available, the other input variables were allowed to be missing.

Our objective was to forecast the AD clinical statuses of participants’ brains – cognitively normal (CN), mild cognitive impairment (MCI) or probable AD – in the next 4 years. Since the amount of the queried data was substantially limited (see Table I), we sampled entries from follow-up examinations to increase the amount of training data, and performed 10-fold cross-validation on this task.

### B. Experimental Setup

#### 1) Implementation Details:

We trained and evaluated our method and the reference approaches using V100 Nvidia GPUs. Each experimental setting was performed on a single GPU with 12GB. We implemented all the methods using the PyTorch framework [45], and trained each of them with the same set of configurations and hyperparameters. For each problem, we used the Adam optimizer [46]. The learning rates of 1e−4 and 1e−5 were set for the OA and AD-related tasks, respectively.

To extract visual representations of 2D images, we utilized the ResNet18 architecture [47] whose weights were pretrained on the ImageNet dataset [48]. We used a batch size of 128 for the knee OA experiments. Regarding 3D images, we chose the 3D-ShuffleNet2 architecture because it was well-balanced between efficiency and performance as shown in [49], which allowed us to train each model with a batch size of 36 on a single consumer-level GPU. We utilized 3D-ShuffleNet2’s weights previously pretrained on the Kinetics-600 dataset [50]. Moreover, we used a common feature extraction architecture with a linear layer, a ReLU activation, and a layer normalization [36] for all scalar numerical and categorical inputs. We provide the detailed description of the input variables in Tables II and III.

#### 2) Baselines:

For fair comparisons, our baselines were models that had the same feature extraction modules for multi-modal data, as described in Section IV-B.1, but utilized different architectures to perform discrete time series forecasting. As such, we compared our method to baselines with the forecasting module using fully-connected network (FCN), GRU [51], LSTM [52], multi-modal transformer (MMTF) [31], Reformer [53], Informer [54], Autoformer [55], or CLIMAT [22]. While FCN, MMTF, Reformer, Informer, Autoformer, and CLIMAT are parallel models, GRU and LSTM are sequential approaches. Among the transformer-based methods both versions of CLIMAT have a modular structure of transformers rather than using a flat structure as in MMTF, Reformer, Informer, and Autoformer.

#### 3) Metrics:

As data from both OAI and ADNI were imbalanced, balanced accuracy (BA) [56] was a must metric in our experiments. As there were only 3 classes in the AD clinical status prognosis prediction task, we also utilized the one-vs-one multi-class area under the ROC Curve (mAUCROC) [57] as another metric. To quantitatively measure calibration, we used expected calibration error (ECE) [35], [58]. We reported means and standard errors of each metric computed over 5 runs with different random seeds.

To perform analyses of the statistical significance of our results, we utilized the two-sided Wilcoxon signed-rank test to validate the advantage of our method compared to each baseline [59]. We equally split the test set into 20 subsets without overlapping patients. For such a subset, we computed metrics averaged over 5 random seeds per method. The statistical testing was done patient-wise by comparing our method with every baseline individually. In the case of the OAI dataset, for all patients, we did two rounds of hypothesis testing: one for the left and one for the right knee, respectively. Subsequently, we applied the Bonferroni correction to adjust the significance thresholds for multiple comparisons (*p* = 0.025 due to two knees per patient) [60].

### C. Ablation Studies

#### 1) Overview:

We conducted a thorough ablation study to investigate the effects of different components in our CLIMATv2 architecture on the OAI dataset. The empirical results are presented in Table IV and summarized in the following subsections.

#### 2) Effect of the Transformer P’s Depth:

Firstly, we searched for an optimal depth of the transformer P. The results show that the transformer P with a depth of 4 provides the best performance, yielding 0.2 % gain in averaged BA compared to depths of 2 and 4. The average BA (over 4 years) indicates a substantial boost in performance. We, therefore, use the depth of 4 for the transformer P in the sequel.

#### 3) Effect of the Number of [CLS] Embeddings and FFNs in the Transformer P:

Then, we simultaneously validated two components: using single or multiple [CLS] embeddings, and using common or separate FFN in the transformer P. Of 4 combinations of settings, the quantitative results suggest that the transformer should have 9 individual [CLS] embeddings, each of which corresponds to an output head, and merely use one common FFN to make predictions at different time points.

#### 4) Effect of the Consistency Term:

To validate the necessity of the ${ℒ}_{\text{cons}}$ term, we conducted an experiment on a set of λ values {0, 0.25, 0.5, 0.75, 1}. The empirical evidence in Table IV shows that a λ of 0.5 resulted in the best performance, which was 0.7% higher than the setting without ${ℒ}_{\text{cons}}$. We further validated the effects of the consistency term on other knee OA grading criteria as well as the AD status forecasting task. The empirical results in Table V consistently demonstrate that the term ${ℒ}_{\text{cons}}$ has a positive impact on performance, albeit with the trade-off of calibration. A consistency coefficient λ of 0.5 is the most optimal setting in terms of performance across the tasks. Specifically, we observed BA gains of 1.7%, 0.6%, and 0.5% with trade-off ECEs of 0.4%, 0.8%, and 0.4% for JSL, JSM, and AD, respectively.

#### 5) Average Pooling for Image Representation:

In contrast to the previous version, we adopted a conventional approach used in prior studies [38], [39], [40], which involves performing an average pooling over the output sequence of the Radiologist block to constitute an imaging feature vector for diagnosis prediction ${\hat{y}}_{0}^{R}$. According to Table IV, such an approach results in a gain of 1.1% BA compared to the baseline, which solely utilized the first vector of the sequence generated by the block R.

#### 6) Multimodal Channel-Wise Concatenation:

We conducted an ablation study on the combination of multimodal embeddings. As such, we compared our channel-wise approach to a sequence-wise baseline that simply concatenates imaging embeddings and a projected version of non-imaging ones. For the baseline, we utilized a linear projection layer to ensure that imaging and non-imaging embeddings are in the same $C_{X}$-dimensional space. We reported the K-fold cross-validation results in Table VI. On the knee OA-related tasks, our approach tends to have positive benefits on both performance and calibration. Specifically, the performance gains were 2.3%, 2.0%, and 0.5% for KL, JSL, and JSM, respectively. Except for JSL with an increase of 0.1% ECE, the approach results in calibration improvements of 2.5% and 3.2 for KL and JSM, respectively. On the AD-related task, the channel-wise approach leads to improvements of 1.1% BA and 0.2% ECE.

#### 7) Effectiveness of CLUB Loss:

**Fig. 6.**
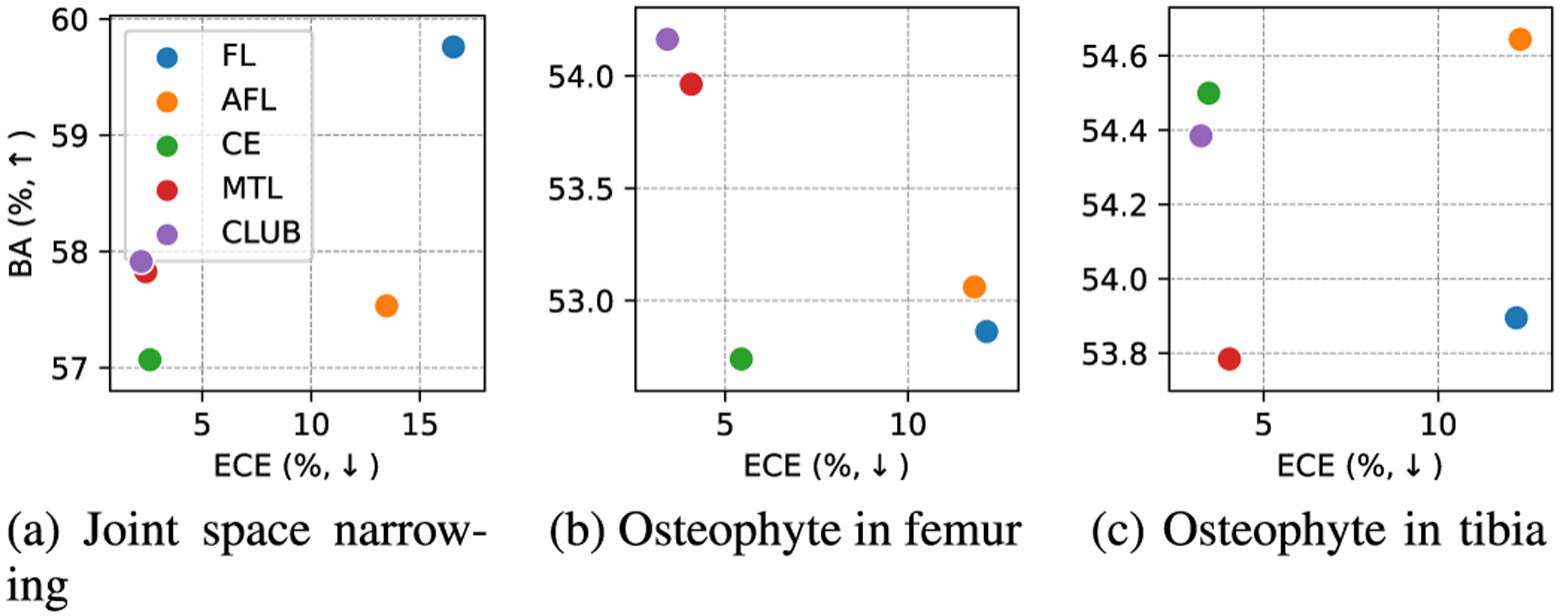
Performance and calibration comparisons between CLUB and other baselines. All the measures are on the medial side. The losses can be categorized into groups: (1) FL and AFL, and (2) CE, MTL, and CLUB, which are based on cross-entropy and focal loss, respectively.

We compared the CLUB loss to CE itself, multi-task loss (MTL) [41], focal loss (FL) [61], and adaptive focal loss (AFL) [62]. Whereas the first two baselines and our loss are based on CE loss, the remaining ones are related to FL. In Figure 6, we graphically visualize the trade-off between performance and calibration, in which the best in both aspects are expected to locate close to the top-left corners. We observe that our model trained with FL-related losses was substantially worse calibrated compared to the settings with any CE-based loss. Among the losses based on CE, the proposed CLUB helped our model to achieve the best ECEs in all three OA grading systems with insubstantial drops in performance.

### D. Performance and Calibration Comparisons to Competitive Baselines

#### 1) Knee OA Structural Prognosis Prediction:

**Fig. 7.**
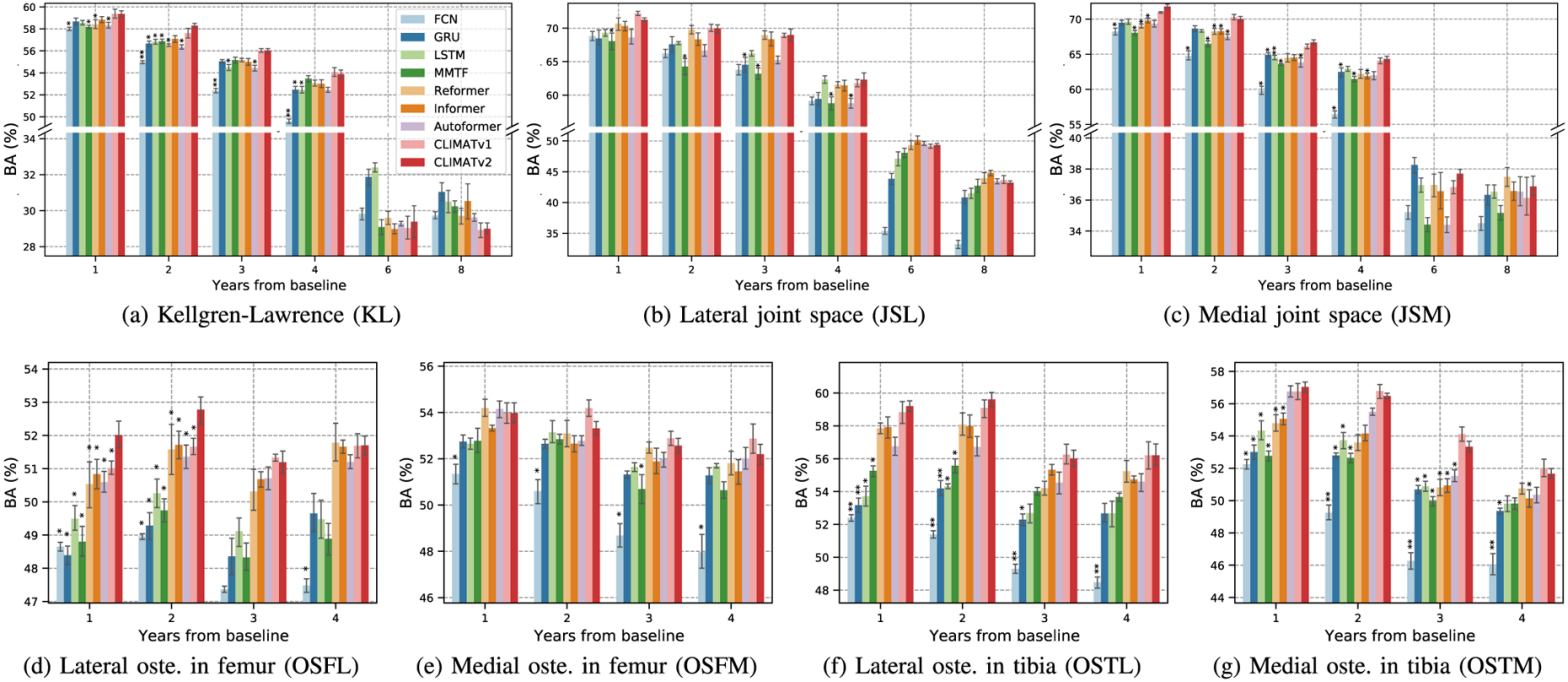
Performance comparisons between our CLIMAT models and other baselines on the knee osteoarthritis prognosis task via different types of grading (means and standard errors over 5 random seeds). * and ** indicate the statistically significant differences between CLIMATv2 compared to each baseline via Wilcoxon signed-rank tests (*p* < 0.05 and *p* < 0.001, respectively). As the statistical tests were conducted on both knees, p-value thresholds were adjusted to 0.025 and 0.0005, respectively.

In Figure 7, we graphically present comparisons between both versions of CLIMAT and the baselines in the 7 different knee OA grading scales.

In general, both versions of CLIMAT outperformed the other baselines in forecasting the knee OA progression within the first 4 years across the knee OA grading systems. That is consistent with the observation of [22] in KL. We observe that LSTM is the most competitive baseline across the grading systems. Compared to LSTM, on average of the first 4 years, our model achieved 1.3%, 1.7%, 1.9%, 2.3%, 0.7%, 4.4%, and 2.4% higher BAs while having 1.9%, 0.1%, 0.02%, 6.5%, 3.6%, 1.4%, and 4.1% lower ECEs in KL, JSL, JSM, OSFL, OSFM, OSTL, and OSTM, respectively. On average, Informer was the most competitive transformer-based baseline. Compared to Informer, CLIMATv2 achieved BA improvements of 0.4%, 0.3%, 1.6%, 0.6%, 0.4%, 1.2%, and 1.8% in KL, JSL, JSM, OSFL, OSFM, OSTL, and OSTM, respectively. Except for KL and OSFM, our model had lower ECEs with differences of 0.3%, 0.1%, 1.5%, 1.2%, and 0.4% in JSL, JSM, OSFL, OSTL, and OSTM, respectively. Moreover, in comparison to CLIMATv1 [22], on average for the first 4 years, the newer version performed better in KL, JSM, OSFL, and OSTL with BA improvements of 0.1%, 0.4%, 0.5%, and 0.2%, respectively, whereas it reached 0.1%, 0.5%, and 0.3% lower BAs in JSL, OSFM, and OSTM, respectively. Regarding the calibration aspect, CLIMATv2 obtained lower ECEs compared to CLIMATv1 in JSL, JSM, OSFM, and OSTM with differences of 0.1%, 0.3%, 0.2%, and 0.4%, respectively.

#### 2) Alzheimer’s Disease Status Prognosis Prediction:

We reported the quantitative results in Table VII. Regarding performance, both the CLIMAT methods achieved the best performances across the prediction targets, in which CLIMATv2 was top-1 at the first 2 years in both BA and mROCAUC. Compared to the transformer-based baseline MMTF, our method outperformed by 2.2%, 1.8%, and 2.2% BAs at years 1, 2, and 4, respectively. In calibration, CLIMATv2 yielded substantially lower ECEs than all the references at every prediction target. That observation was supported by the statistical test results in Table VII.

### E. Attention Maps Over Multiple Modalities

The self-attention mechanism of the transformers in CLIMATv2 allowed us to visualize attention maps over imaging and non-imaging modalities when our model made a prediction at a specific target. Specifically, we used Softmax ($\left(Q_{L}K_{L}^{⊺}∕\sqrt{d_{k}}\right)$), where $Q_{L}$, $K_{L}$ are query and key matrices of the last layer L, respectively, and $d_{k}$ is the feature dimension of the key matrix, as attention maps [25]. While we utilized the softmax output corresponding to ${\bar{h}}_{C}^{0}$ in the transformer F for clinical variables, we took the softmax output in computing ${\bar{h}}_{P}^{t}$ with t=0,…,T in the transformer P to visualize attention maps on imaging modalities. Here, we set t=1, corresponding to the forecast of a disease severity 1 year from the baseline.

#### 1) Knee OA Structural Prognosis:

**Fig. 9.**
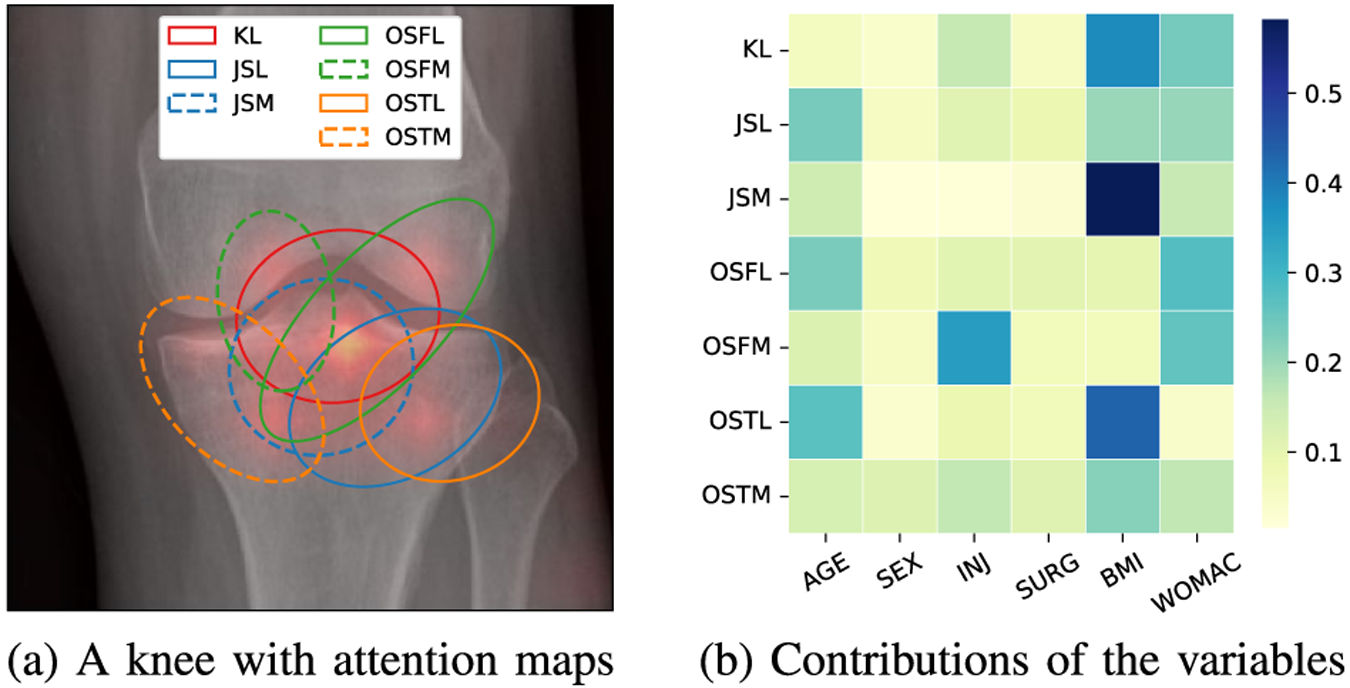
An example of progression from a healthy knee at baseline to early osteoarthritis. Our model identified the changes in the intercondylar notch, sex, and symptomatic status.

In Figure 9, we visualized attention maps over different input modalities across 7 grading criteria. As such, in Figure 9a, we displayed a healthy knee at the baseline overlaid by 7 corresponding saliency maps. For differentiation, we also provided colored ellipses. Figure 9b shows the heatmap over the 6 clinical variables on each grading criterion. Values on each row sum up to 1. In this particular case, we observe that the model has paid the most attention to the intercondylar notch, together with BMI and WOMAC [63].

#### 2) AD Clinical Status Prognosis:

**Fig. 10.**
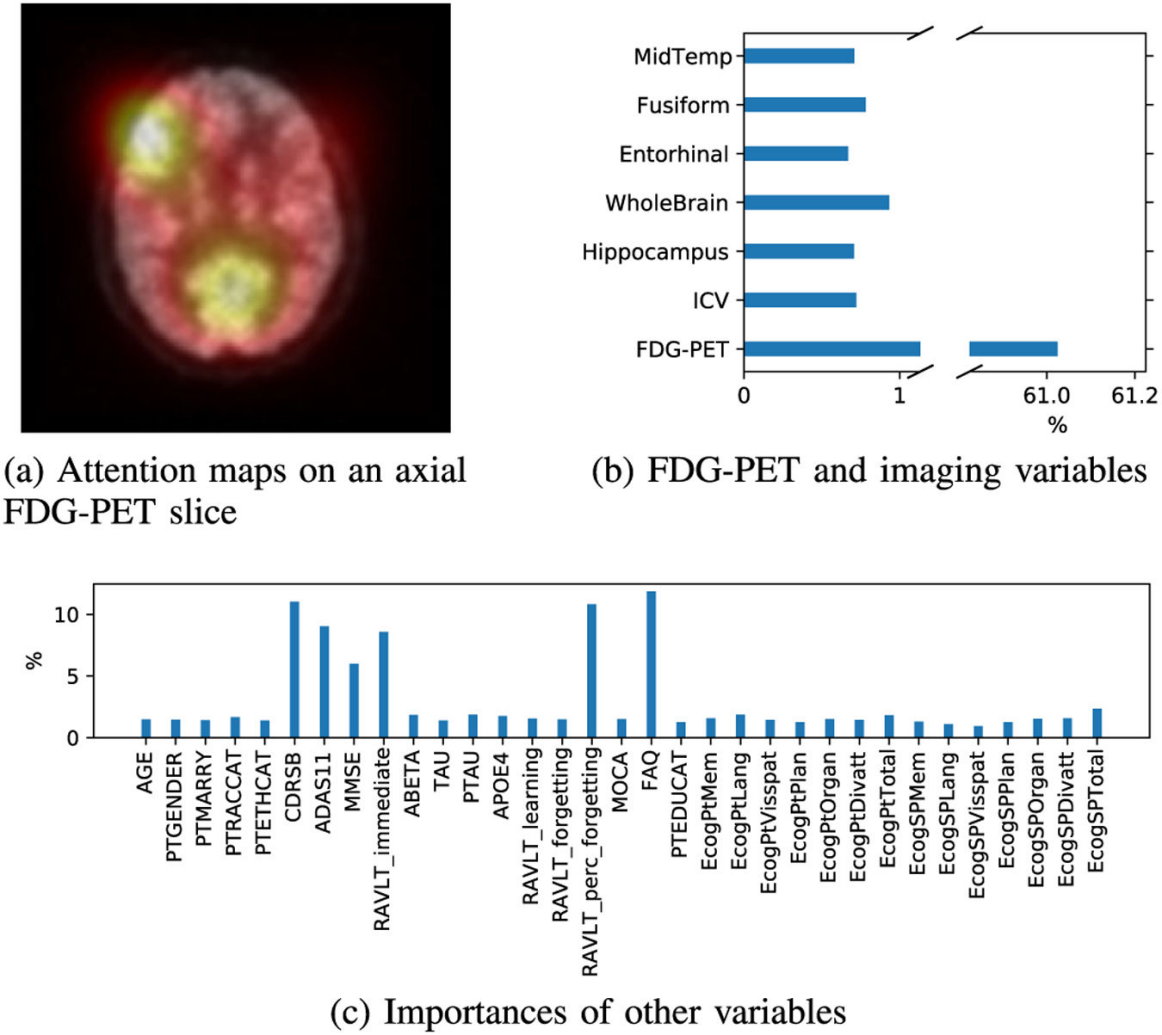
Interpretability of our method’s prediction on a selective sample from the ADNI dataset.

As imaging data consisted of 3D FDG-PET scans as well as the other imaging measurements, we had to separate them into Figures 10a and 10b. We can observe that an attention sphere locates around the posterior cingulate cortex, the inferior frontal gyrus, and the middle gyrus [64]. Figure 10b shows accumulated attention weights corresponding to the FDG-PET feature vectors alongside ones of the other imaging measurements. The reason that imaging variables were assigned a substantially higher importance is that the number of the 3D visual embeddings was dominant compared to the others (i.e. 125 versus 6). In Figure 10c, high attention can be observed on the percent forgetting score of the Rey Auditory Verbal Learning Test (RAVLT), RAVLT immediate, the AD assessment score 11-item (ADAS11), Clinical Dementia Rating Scale-Sum of Boxes (CDRSB), Mini-Mental State Exam (MMSE), and Functional Activities Questionnaire (FAQ).

## V. Conclusion

In this paper, we proposed a novel general-purpose transformer-based method to forecast the trajectory of a disease’s stage from multimodel data. We applied our method to two real-world applications, that are related to OA and AD. Our framework provides tools to integrate multi-modal data and has interpretation capabilities through self-attention.

In comparison with the prior version, CLIMATv2 has two primary upgrades. First, we have eliminated the assumption of independence between non-imaging data $m_{0}$ and diagnostic predictions $y_{0}$ used in CLIMATv1 [22] since it does not hold not only in OA and AD, but also in other diseases. Specifically, Liu et al. [34] provided empirical evidence of the benefit of the inclusion of non-imaging data in the knee OA grading task. The study conducted by Bird et al. [65] indicated a link between human genes and AD while Li et al. [66] showed that a blood test can detect the existence of amyloid-beta plaques in the human brain, which is strongly associated with AD status. Second, we have proposed the CLUB loss, which allowed us to optimize for both performance and calibration.

There are some limitations in this study, which are worth mentioning. First, we used common DL architectures as imaging and non-imaging feature extractors. While such a standardized procedure resulted in fair comparisons, better results could have been obtained with e.g. Neural Architecture Search methods [67]. Furthermore, a wider range of DL modules could have been considered, but this could substantially increase the use of computing resources. Specifically, to obtain results in this work, it required roughly 400 GPU hours for experiments in Table VII and 525 GPU hours in Figure 7 for every method, respectively.

**Fig. 11.**
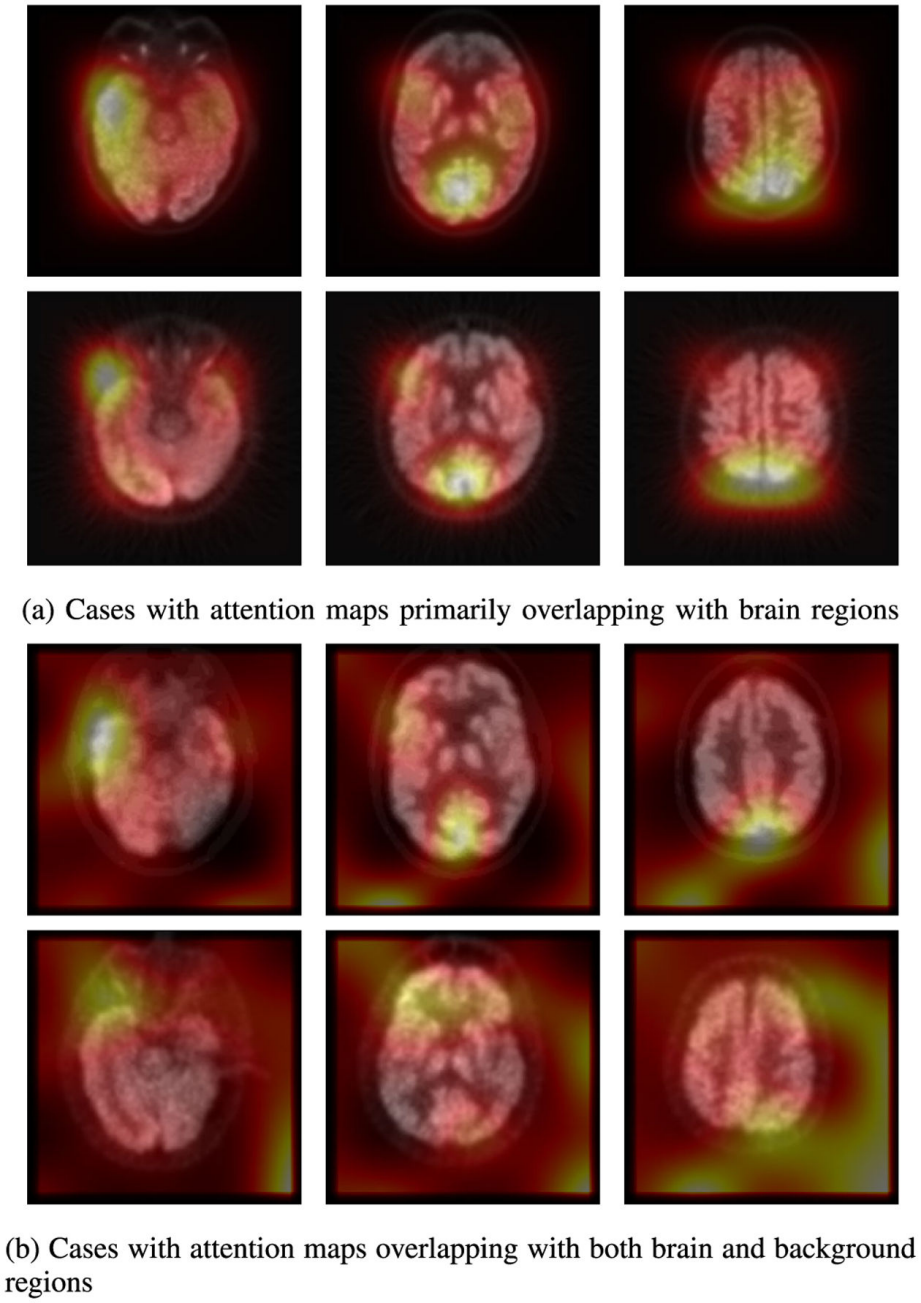
Attention maps on axial FDG-PET slices. Three axial slices on each row belong to the same PET scan.

The second limitation of the present study, is that attention maps produced by transformers act as human-friendly signals of our model, and should be carefully used in practice with expert knowledge in the domain. Transformers may highlight areas not associated with the body part, which can be seen in Figure 11 as well as in other studies [68], [69], [70].

Lastly, we primarily utilized the transformer proposed by [27]. More efficient and advanced transformers such as [53], [54], [55], and [71] could be further investigated to integrate into the framework.

To conclude, to our knowledge, this is not only the first study in the realm of OA, but also the first work on AD clinical status prognosis prediction from the multi-modal setup that includes raw 3D scans and scalar variables. The developed method can be of interest to other fields, where forecasting of calibrated disease trajectory is of interest. An implementation of our method is made publicly available at https://github.com/Oulu-IMEDS/CLIMATv2.

## Figures and Tables

**Fig. 3.**
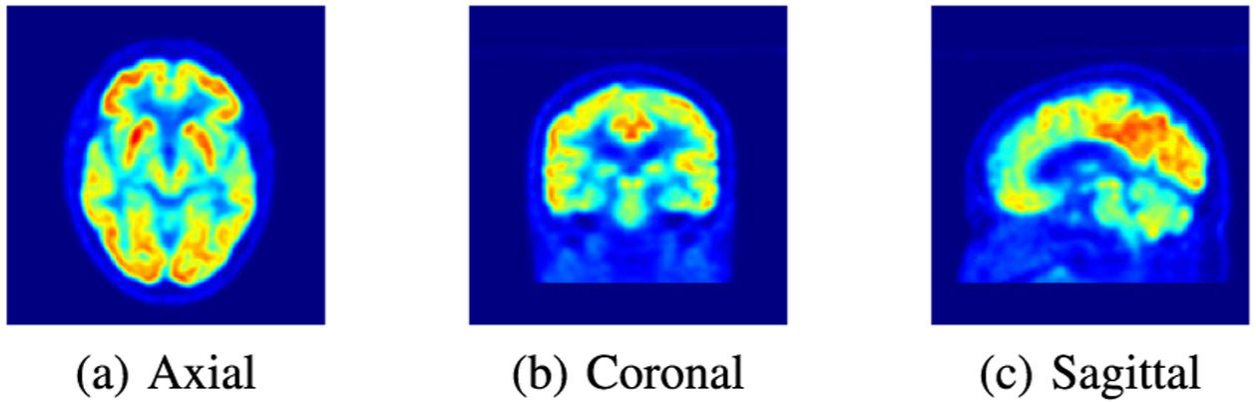
The three projections of a 3D FDG-PET scan, which is converted to the jet colormap for demonstration purposes. The red regions are associated with high concentrations of the FDG radioactive tracer in the brain.

**Fig. 8.**
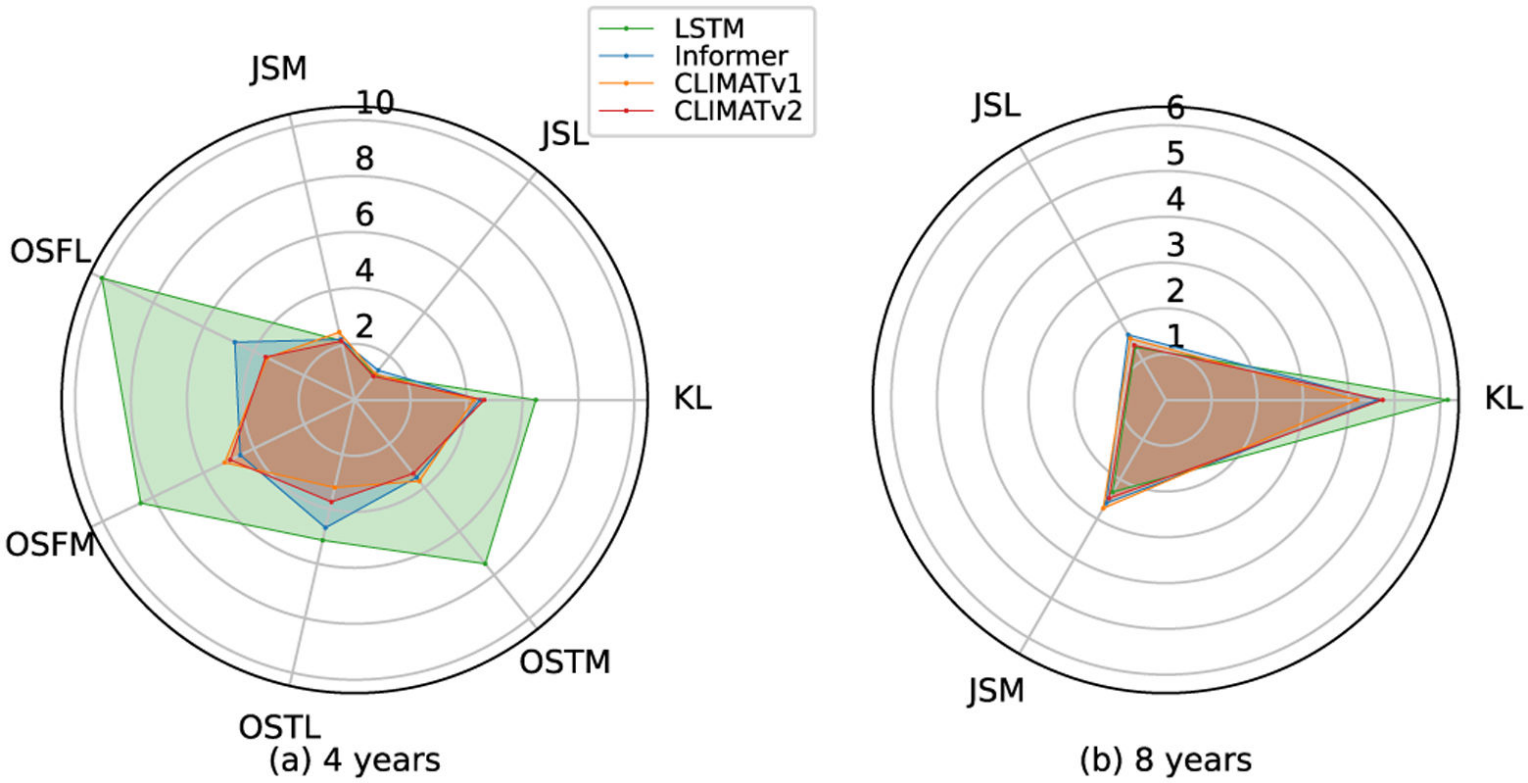
Calibration comparisons on the knee OA prognosis predictions. (a) Averaged ECEs over the first 4 years. (b) Averaged ECEs over 8 years.

**TABLE I:** Dataset Statistics. Subjects Are Patient Knee joints, and Patient brains for OAI, and ADNI, Respectively

Dataset	Task	# subjects
OAI	OA structural prognosis	8,953
ADNI	AD clinical status prognosis	1,307

**TABLE II:** Input Variables for Forecasting Knee OA Severity Grades

Group	Variable name	Data type
Raw imaging	Knee X-ray	2D
Clinical Variables	Age	Numerical
	WOMAC	Numerical
	Sex	Categorical
	Injury	Categorical
	Surgery	Categorical
	BMI	Numerical

**TABLE III:** Input Variables for Forecasting Clinical Statuses of Alzheimer’s Disease. See Sec. IV-E.2 for Acronyms

Group	Variable name	Data type
Raw imaging	FDG-PET	3D
MRI measures	Hippocampus	Numerical
	Whole brain	Numerical
	Entorhinal	Numerical
	Fusiform gyrus	Numerical
	Mid. temp. gyrus	Numerical
	Intracranial vol.	Numerical
Clinical variables	Sex	Categorical
	Marriage	Categorical
	Race	Categorical
	Ethnicity	Categorical
	Education	Numerical
Cognitive tests	CDRSB	Numerical
	ADAS11	Numerical
	MMSE	Numerical
	RAVLT	1D
Cerebrospinal fluid	A-Beta	Numerical
	Tau	Numerical
	Ptau	Numerical
	Moca	Numerical
	Ecog	1D
Risk factors	Apolipoprotein E4	Numerical
	Age	Numerical

**TABLE IV:** Hyperparameter and Model Selection Based on CV Performances on the KL-based Knee OA Prognosis Prediction Task. BA* Indicates the Averages of BAs of the Targets at the Baseline and the First 4 Years

Depth	# of [CLS]	FFN_0:*T*_	λ	Image rep.	BA* (%)
2	9	Separate	0.5	Average pool	59.2
**4**					**59.4**
6					59.2
4	1	Common	0.5	Average pool	58.3
	**9**	**Common**			**59.8**
	1	Separate			59.7
	9	Separate			59.4
4	9	Common	0.0	Average pool	59.1
			0.25		58.9
			**0.5**		**59.8**
			0.75		56.1
			1.0		56.7
4	9	Common	0.5	**Average pool**	**59.8**
				[CLS] head	58.7

**TABLE V:** Effect of the Consistency Term on Performance and Calibration (K-Fold Cross-Validation). Reported Results Are Averages of BAs and ECEs Over the First 4 Years

Grading		JSL		JSM		AD	
	λ	BA	ECE	BA	ECE	BA	ECE
Without ${ℒ}_{\text{cons}}$	0	63.2	1.4	64.7	8.8	86.8	7.5
With ${ℒ}_{\text{cons}}$	0.25	63.1	1.5	64.5	8.7	86.8	8.3
	0.50	64.9	1.8	65.3	9.6	87.3	7.9
	0.75	64.8	1.8	65.1	9.5	87.3	8.1
	1	64.6	1.7	65.2	9.5	86.7	9.2

**TABLE VI:** Ablation Study on Imaging and non-imaging Combination With k-Fold Cross-Validation (*K* = 5 and *K* = 10 for OAI and ADNI, Respectively). Channel-Wise Approach (Ours) Is Compared to the Sequence-Wise Approach, Concatenating Imaging Embeddings Produced by the Block R With Projected Non-Imaging Embeddings Outputted by the Block C. Reported Results Are Averaged BAs and ECEs Over the First 4 Years

Grading	Setting	BA (%, ↑)	ECE (%, ↓)
KL	Channel-wise	**59.8**	**14.7**
	Sequence-wise	57.5	17.2
JSL	Channel-wise	**64.9**	1.8
	Sequence-wise	62.9	**1.7**
JSM	Channel-wise	**65.3**	**9.6**
	Sequence-wise	64.7	12.8
AD	Channel-wise	**87.3**	**7.9**
	Sequence-wise	86.2	8.1

**TABLE VII:** CV Performance and Calibration Comparisons on the ADNI Data (Mean and Standard Errors Over 5 Random Seeds). The Best Performances With and Without Substantial Differences Are Indicated by Bold and Underlined Values, respectively. The Substantial Improvement Is Determined by Whether the Best Performance Overlaps With Any Other method’s. * and ** Indicate the Statistically Significant Differences Between CLIMATv2 vs. Each Baseline via Wilcoxon signed-Rank Tests (P < 0.05 and ****p < 0.001, Respectively)

Year	Method	BA (%, ↑)	mROCAUC (%, ↑)	ECE (%, ↓)
1	FCN	87.6±0.2**	96.6±0.1**	9.5±0.3**
	GRU	87.1±0.3**	96.6±0.1**	8.6±0.4*
	LSTM	87.9±0.2**	96.8±0.1**	8.7±0.3**
	MMTF	88.2±1.0	96.4±0.8*	23.3±0.4**
	Reformer	80.8±1.0**	93.6±0.7**	9.6±0.3**
	Informer	78.4±1.5**	93.1±0.6**	10.0±0.4**
	Autoformer	85.2±0.3**	96.0±0.1**	7.8±0.2*
	CLIMATv1	90.1±0.1	97.6±0.1*	6.7±0.2
	CLIMATv2	**90.4±0.1**	**98.0±0.1**	**6.5±0.1**
2	FCN	85.5±0.2*	95.7±0.1*	9.2±0.2*
	GRU	85.1±0.2*	95.6±0.1*	9.7±0.2*
	LSTM	85.7±0.3*	95.7±0.2*	9.3±0.7
	MMTF	85.4±0.6	95.3±0.7	22.3±0.4**
	Reformer	78.8±0.9**	92.5±0.6**	9.2±0.2*
	Informer	78.1±1.3**	92.9±0.4**	8.6±0.1
	Autoformer	83.7±0.3**	95.2±0.1**	8.6±0.2
	CLIMATv1	87.2±0.0	96.6±0.0	9.1±0.2*
	CLIMATv2	87.2±0.2	96.6±0.1	**7.9±0.3**
4	FCN	80.7±0.3*	93.7±0.1	9.6±0.3*
	GRU	81.7±0.1	93.8±0.1	12.6±0.4**
	LSTM	81.0±0.7*	93.5±0.2	12.4±0.6**
	MMTF	80.6±0.7	93.0±0.6	17.9±0.3**
	Reformer	76.7±0.9**	90.5±0.5**	11.1±0.5*
	Informer	71.6±0.9**	87.4±0.3**	12.6±0.3**
	Autoformer	80.5±0.3**	93.3±0.1**	9.5±0.2
	CLIMATv1	83.0±0.2	**94.5±0.1**	9.6±0.3*
	CLIMATv2	82.8±0.1	94.2±0.1	**9.2±0.2**

## Footnotes

1 In the sequel we write general practitioner, which, however, does not restrict our modeling approach.

2 Hereinafter, we use the terms *block* and *transformer* interchangeably.
